# The Intestinal Barrier and Current Techniques for the Assessment of Gut Permeability

**DOI:** 10.3390/cells9081909

**Published:** 2020-08-17

**Authors:** Ida Schoultz, Åsa V. Keita

**Affiliations:** 1 Faculty of Medicine and Health, School of Medical Sciences, Örebro University, 703 62 Örebro, Sweden; ida.schoultz@oru.se; 2 Department of Biomedical and Clinical Sciences, Linköping University, 581 85 Linköping, Sweden

**Keywords:** intestinal barrier, gut permeability, paracellular route, techniques, transcellular route, paracellular probes

## Abstract

The intestinal barrier is essential in human health and constitutes the interface between the outside and the internal milieu of the body. A functional intestinal barrier allows absorption of nutrients and fluids but simultaneously prevents harmful substances like toxins and bacteria from crossing the intestinal epithelium and reaching the body. An altered intestinal permeability, a sign of a perturbed barrier function, has during the last decade been associated with several chronic conditions, including diseases originating in the gastrointestinal tract but also diseases such as Alzheimer and Parkinson disease. This has led to an intensified interest from researchers with diverse backgrounds to perform functional studies of the intestinal barrier in different conditions. Intestinal permeability is defined as the passage of a solute through a simple membrane and can be measured by recording the passage of permeability markers over the epithelium via the paracellular or the transcellular route. The methodological tools to investigate the gut barrier function are rapidly expanding and new methodological approaches are being developed. Here we outline and discuss, in vivo, in vitro and ex vivo techniques and how these methods can be utilized for thorough investigation of the intestinal barrier.

## 1. Introduction

The increased attention of the concept “leaky gut” and its association with numerous gastrointestinal (GI) disorders has led to an intensified interest from researchers with diverse backgrounds to perform functional studies of the intestinal barrier in different conditions. The methodological tools to investigate the intestinal barrier function are rapidly expanding and new methodological approaches are being developed continuously. For research groups new to the field of intestinal functional studies the most appropriate technique might therefore be difficult to identify. Gut barrier function involves the regulation of translocating luminal content such as antigens and bacteria that pass through the epithelial cell layer either between the epithelial cells (paracellular route) or through the cells (transcellular route) into the underlying mucosa. Intestinal permeability can be measured by recording the passage of permeability markers over the epithelium via these passage routes [1]. An increased intestinal permeability and signs of a dysfunctional barrier have been observed in several different conditions, such as Parkinson disease [2], obesity [3] and diabetes type 2 [4], as summarized in Table 1.

Many of the conditions associated with a leaky gut show signs of both increased paracellular and transcellular permeability. Moreover, new exciting findings connecting different conditions to a dysfunctional barrier by primarily in vivo biomarkers could potentially be complemented with other techniques to strengthen these findings.

To guide researchers new to functional studies of the intestine we outline how paracellular and transcellular permeability can be assessed in vivo, in vitro and ex vivo. Although, markers of intestinal inflammation or gut microbiota composition are not the focus of this review it is important to acknowledge that these factors play an essential role in gut barrier homeostasis and are important complements in studies of intestinal barrier function, particularly when investigating the underlying mechanisms of a perturbed intestinal barrier.

## 2. The Intestinal Barrier

**Figure 1:**
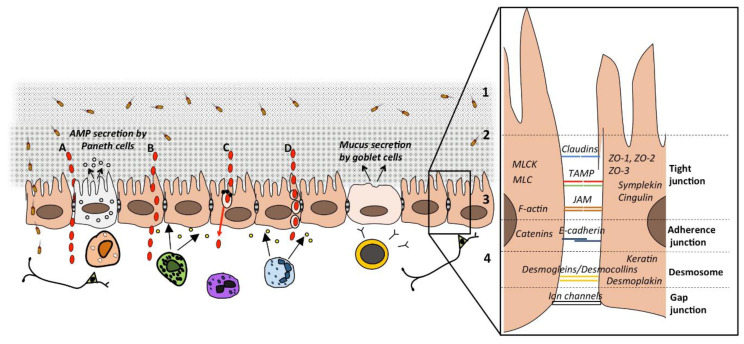
A schematic drawing of the intestinal barrier and passage routes across the epithelium. Solutes can pass the intestinal epithelium via either the (**A**) paracellular route (larger hydrophilic solutes); (**B**) transcellular route (small hydrophilic and lipophilic solutes) (**C**); transcellular route via aqueous pores (small hydrophilic solutes) or active carrier-mediated absorption (nutrients); or (**D**) endocytosis, followed by transcytosis and exocytosis (larger particles, peptides and proteins). The barrier constitutes of (1) the lumen, bacteria and antigens are degraded by biliary juices, gastric and pancreatic acids and the colonization of pathogens is inhibited by commensal bacteria producing antimicrobial substances; (2) the microclimate; unstirred water layer, glycocalyx, bacterial adhesion is prevented by mucus and IgA secretion; (3) the epithelial cells; luminal content is transported while noxious stimuli is impeded by chloride secretion and production of antimicrobial peptides (AMP), junctional complexes between the cells regulate permeability, for details see right panel; (4) the lamina propria; immunoglobulins and cytokines are secreted from cells of both the innate and acquired immunity with direct or indirect effects on permeability, interactions with the endocrine and enteric nervous system. TAMP: tight junction-associated-MARVEL proteins including occludin, tricellulin and Marvel D3; JAM: junctional adhesion molecule; MLC: myosin light chain; MLCK: MLC of myosin II kinase.

The intestinal barrier constitutes the interface between the outside and the internal milieu. A functional intestinal barrier allows absorption of nutrients and fluids but simultaneously prevents harmful substances like toxins and bacteria from passing through the intestinal epithelium to the underlying tissue [30]. There is a delicate balance to keep a functional barrier and it is maintained by physical defense mechanisms including both the junctional complexes linking adjacent epithelial cells and the mucosal surface of the epithelial cell lining (Figure 1).

To maintain a functional barrier, several mechanical properties of the intestinal epithelial cells are essential and constitute various defense mechanisms, where the first one is the lumen itself. Here, antigens and bacteria are degraded by biliary juices and gastric and pancreatic acids. Commensal luminal bacteria inhibit the colonization of pathogens by for example the production of bacteriocins, pH modification of the luminal content, and competition for nutrients required for growth of pathogens. The commensal flora is continuously in contact with the intestinal epithelium and has been implicated to shape the intestinal barrier structure by for example inducing physiological paracellular permeability essential for nutrient uptake and strengthening of the mucus layer [31,32]. The next defense mechanism is the microclimate that includes the unstirred water layer, the glycocalyx, and the mucus layer [33]. The mucus layer forms a protected habitat for the commensal flora in close proximity to the epithelial cells [34,35]. The mucus is composed of mucin produced and secreted by the goblet cells. In addition, the mucus layer consists of IgA, one of the most abundant antibodies in mucosal secretions, that through several different mechanisms neutralize pathogenic bacteria and favor the maintenance of the commensal flora [36].

The next part of the intestinal barrier is the epithelium, the single cell layer that separates the body from the external luminal milieu. The epithelium is composed of several different cell types, such as enterocytes, Paneth cells and goblet cells. These cells and their different functions form a tight barrier towards the intestinal luminal milieu [37]. Paneth cells produce antimicrobial peptides that contribute to the clearance of pathogenic bacteria [38], while enterocytes respond to noxious stimuli with chloride secretion. Underneath the intestinal epithelium is the lamina propria consisting of innate and adaptive immune cells, such as neutrophils, T-regulatory cells, macrophages and mast cells. These cells react immediately in response to the invasion of foreign substances and act together to clear the inflammation. Neutrophils are among the first cells reaching inflamed areas and limit the invasion of microorganisms by eliminating them via phagocytosis [39]. Likewise, macrophages in the lamina propria reside close to the enterocytes and phagocyte potential harmful luminal content that have breached the intestinal barrier and reached the lamina propria [40]. T-regulatory cells are critical in the maintenance of immune homeostasis as they are able to suppress the activation of various immune cells involved in intestinal inflammation as well as inducing immune tolerance to antigens derived from the diet or commensal flora [41]. The lamina propria also holds the endocrine- and enteric nervous system that plays an important role in gut barrier homeostasis. Mast cells are located close to nerves and can be activated by neuronal mediators and have been implicated in several types of neuro-inflammatory responses as reviewed by Keita et al. [42].

The gut barrier homeostasis is dependent on the relationship between the gut microbiota and the intestinal epithelium. The innate immune system and particularly the pattern-recognition receptors (PPRs) expressed in enterocytes (i.e., Toll-like receptors, nucleotide oligomerization domain like receptors and retinoic acid inducible gene I) are essential for the homeostasis of the gut [43]. These receptors recognize microbial signature molecules, so called microbial/pathogen-associated molecular patterns (MAMPs/PAMPs) expressed by most microbes [44]. The attachment of MAMPs/PAMPs to the PPRs elicits an immediate inflammatory response against foreign microorganisms to protect the host [45]. This interaction enables the identification of foreign molecules by antigen presenting cells, such as dendritic cells and macrophages. These cells further migrate to the peripheral site where they present antigens to T-cells leading to the production of pro-inflammatory cytokines, such as interferon gamma (IFN-γ), chemokines and antimicrobial peptides [46] to protect the intestinal barrier. One of the major signaling cascades elicited by IFN-γ and which is involved in maintaining the intestinal barrier is activation of the tryptophan metabolism by inducing increased levels of the enzyme indoleamine 2,3-dooxygenase [47]. This enzyme is responsible for the conversion of tryptophan to kynurenine which functions as an endogenous ligand of the aryl hydrocarbon receptor (AhR) transcription factor [48] that upon activation have the ability to inhibit inflammatory responses in the gut and have a protective effect against IBD [49].

During normal circumstances the symbiosis between the gut microbiota and host is well balanced. However, in certain diseases such as IBD this equilibrium is broken and contribute to intestinal inflammation [50]. In addition, a dysbiosis of the gut microbiota has been associated with several diseases associated with a leaky gut such as diabetes, depression and Alzheimer’s disease as reviewed by Luca et al. [51]. These conditions have particularly been associated with a reduced number of strains belonging to the commensal flora and known to exert many beneficial effects, such as *Faecalibacterium (F.) prauznitzii*. *F. prauznitzii* is essential for the fermentation of non-digestible substrates like dietary fibers and endogenous intestinal mucus [52]. The fermentation process supports the growth of microbes specialized in producing short chain fatty acids such as butyrate [52]. Butyrate is the main energy source for human colonocytes and is essential for homeostasis in the intestinal epithelium [53]. Potentially, leading to a diminished intestinal barrier function as experiments in cultured epithelial cells indicate a role of butyrate in the improvement of the intestinal barrier function [54,55]. Hence, assessing the gut microbiota composition, microbial metabolites, as well as inflammatory markers can be important to map the mechanisms behind a perturbed intestinal barrier.

## 3. Intestinal Permeability

A crucial function of the intestinal epithelium is the maintenance of a proper barrier function, allowing the permeability of nutrients, water and ions, but limits entry of pathogens and bacterial toxins. Intestinal permeability is defined as the non-mediated intestinal passage of medium-sized hydrophilic molecules occurring towards a concentration gradient without the assistance of a carrier system [56]. Hence, an increased intestinal permeability is a sign of a perturbed intestinal barrier function. Since the definition of intestinal permeability refers to the passage of a solute through a simple membrane, and the intestinal membrane consists of several layers and different cell types, it is compulsory to use simplifications when measuring intestinal permeability. Intestinal permeability can be assessed via measurements of the transepithelial resistance (TER) i.e., the ability for passive diffusion of ionic charge across the epithelia, but also by measuring passage of solutes over the epithelium [57] via different passage routes.

## 4. Epithelial Passage Routes

Solutes can pass across the intestinal epithelium either between the cells via the paracellular route or through the cells via the transcellular route as shown in Figure 1. Passage via the transcellular route can occur in different ways, depending on the properties of the solute. Alterations in how peptides pass through the epithelium are believed to be of great importance in the pathophysiology of GI disorders.

### 4.1. The Paracellular Route

The paracellular route represents the passage between the cells, via the tight junctions and intercellular spaces [58]. This route is used by medium-sized (≤600 Da in vivo; ≤10 kDa in vitro in cell lines) hydrophilic molecules and normally, the paracellular route is impermeable to protein-sized molecules and thus constitutes an effective barrier to antigenic macromolecules. The epithelial cells are joined to each other by junctional complexes consisting of tight junctions, adherens junctions, desmosomes and gap junctions [59], as illustrated in Figure 1. Tight junctions, also called zonula occludens, are located at the apical part of the lateral membrane forming a network of linking strands. They are important in epithelial transport towards and away from the lumen and in maintaining the polarity of the epithelial cells [60]. Tight junctions appear as multiprotein complexes embedded into the plasma membrane that interact with the adjacent cell. The tight junction complex consists of transmembrane proteins including occludin [61], tricellulin [62] and Marvel D3 [63], all belonging to the tight junction-associated-MARVEL proteins (TAMP) as well as claudins [64] and members of the junctional adhesion molecule (JAM) protein family [65]. The human claudin family includes over 20 members [66] and the distribution of these varies in different tissues [60]. Tricellulin, mainly located at contact points of three cells [62], forms a central tube in tricellular junctions that allows passage of large solutes (≤10 kDa). In cultured epithelial cells, the amount of tricellulin expression regulates macromolecular permeability [62]. There is a size and charge-selectivity within the tight junction permeability barrier, where ions and positively charged molecules pass more easily. The tight junction complex is connected to the cytoskeleton of the adjacent cells via the scaffolding proteins ZO-1, ZO-2 and ZO-3 but also several peripheral proteins like cingulin and symplekin [60]. Further, myosin light chain of myosin II kinase (MLCK) phosphorylates myosin light chain (MLC), affecting the actin cytoskeleton, a process which is essential and critical for the regulation of paracellular permeability [67].

Below the tight junctions are the adherens junctions, constituted of molecules belonging to the cadherin family. Together the adherence junctions and tight junctions form one single functional unit [68], the apical junctional complex. This complex is linked to the cytoskeleton via the perijunctional F-actin ring [69]. Between the epithelial cells, most often below the adherens junctions, are the spot-like dense adhesions called desmosomes located. The desmosomes are constituted of the desmoglein and desmocollin families of desmosomal cadherins and connecting proteins such as desmoplekin and keratin. Finally, are the gap junctions, intercellular channels allowing ions and small molecules to pass between cells, thus linking the interior of adjacent cells.

Measurements of TER are sometimes referred to as paracellular permeability. TER correlates with the free movement of ions and solutes across the epithelium [57] and can be referred to as the paracellular integrity of the tissue, however, it is of importance to note that TER cannot be equated to paracellular permeability of paracellular markers. During experiments, it is not unusual that TER is unchanged while paracellular permeability is increased, or vice versa. This probably refers to different regulations of permeability pathways, i.e., the leak and the pore pathways as described by Shen et al. in 2011 [70].

### 4.2. The Transcellular Route

The transcellular route is the passive diffusion through the cells, used by lipid soluble and small hydrophilic compounds. In addition, active and energy-dependent uptake takes place through the cell as well. Large particles and molecules, like proteins and bacterial products that cannot pass through the cell membrane or the paracellular space, can be taken up via endocytosis, i.e., by the cell through invagination of the plasma membrane followed by vesicle formation. The passage of bacteria and bacterial products are particularly investigated together with paracellular markers in studies focusing on intestinal barrier function. Endocytosis mediates uptake of foreign antigens against which the body can initiate an appropriate immune response. Following endocytosis, the engulfed substances are actively transported through the cytoplasm by transcytosis, which is essential for antigen surveillance in the GI tract [71]. Endocytosis and transcytosis are pathways manipulated by foreign microbes to establish host entry and an intact barrier rely on the correct function of these pathways and that the ability of the cell to eliminate foreign substances. Endocytosis in epithelial cells can occur differently depending on the nature of the substance that is taken up.

The first route is via clathrin-mediated endocytosis, a highly specific receptor-mediated process, which is utilized mainly by immunoglobulins and viruses. Molecules that have bound specifically to the cells get internalized and clathrin-coated vesicles are formed [72]. The second route is by phagocytosis, used by bacteria, viruses, and particles [73] and involves binding of molecules to the cells via receptors. Phagocytosis is relevant for the uptake of antigens derived from bacteria and the diet, and the process is triggered by secreted solubles from invading bacteria [74]. The third route, micropinocytosis, is a non-specific process by which extracellular fluid can be internalized, along with dissolved molecules and viruses, bacteria and apoptotic cell fragments. Micropinocytosis is initiated through invagination of the cell membrane with the formation of circular ruffles that are released in the cytoplasm as a vesicle, a so called macropinosome [75]. The fourth route is via lipid rafts/caveolae, which involves a flask-shaped invagination of cholesterol-enriched microdomains within the plasma membrane that may contain a coat protein, caveolin [76]. Studies have shown that certain enterotoxins and viruses may be endocytosed via rafts/caveolae into enterocytes [77].

## 5. Modulation of the Passage Routes

In many studies it is of interest to verify which route a specific probe or bacteria utilize to pass through the epithelium. Several compounds are known to modulate both the paracellular and transcellular route as outlined below.

### 5.1. Paracellular Route

There are several stimuli known to affect the cytoskeleton and thereby induce changes in paracellular permeability (Figure 1) [78,79]. This is mainly mediated via phosphorylation of MLC by MLCK which affect the F-actin fibers in the perijunctional F-actin ring [80]. Particularly, IFN-γ, TNF and enteropathogenic *Escherichia coli* (EPEC) are known to induce an MLCK-dependent increase in paracellular permeability [81,82].

This pathway is also involved in the physiological regulation of uptake of nutrients, such as glucose, where the activation of the Na^+^-glucose cotransporter sodium-glucose cotransporter (SGLT−1) induces MLCK mediated phosphorylation of MLC [83]. Cytokines, like IFN-γ and TNF, often induce rapid changes without affecting the expression of the tight junction proteins [84], while others like IL−13 induce an increased paracellular permeability by affecting the expression of claudin−2 [85]. Apart from acting via MLCK other pathways are essential in the cytokine regulation of tight junctions. TNF-induced apoptosis has been proposed as an additional pathway involved in the paracellular permeability triggered by this cytokine [86,87]. In addition, IFN-γ induces an activation of the small GTPase Rho A, involved in the regulation of the perijunctional F-actin ring, and the induction of the increased expression of the Rho associated kinase (ROCK), which phosphorylates and activates MLC [88]. Together these actions result in the disassembly of the tight junction complex and consequently elevated paracellular permeability. Similarly, *Clostridium difficile* induce a compromised intestinal barrier by inhibiting the Rho GPTases [89]. A more detailed review of the regulation of the tight junction and paracellular route have been made by Shen [78] and Buckley and Turner [79]. The complexity of the regulation of the tight junction and paracellular route makes it difficult to investigate in functional studies. Specific inhibitors of MLCK are, however, available and have successfully been used in ex vivo Ussing chamber experiments [90] and in vitro cell culture studies [81] to elucidate if specific compounds or conditions induce MLCK-dependent paracellular permeability.

### 5.2. Transcellular Route

To confirm that the passage is via the transcellular route, and also define sub-routes of this pathway (Figure 1B–D), there are several substances to use in vitro and ex vivo, however, the specificity of them varies. 

A common endocytosis/transcytosis inhibitor is dynasore, which inhibits dynamin, i.e., the GTPAse protein enabling movement of the vesicles along the cytoskeleton [91]. A more specific inhibitor for caveolin-mediated endocytosis is the cholesterol-binding agent filipin [92], which binds to cell surface cholesterol and thereby inhibits endocytosis. For clathrin-mediated endocytosis, chlorpromazine is a good candidate, which prevents the assembly and disassembly of clathrin lattices on endosomes and on cell surfaces [93]. There are also more general inhibitors that can be used for studies on endocytosis, such as colchicine, which disrupts vesicular trafficking and formation [94], and cytochalasin D, which inhibits actin polymerization. To read more about these and other endocytosis inhibitors authors refer to [95].

## 6. Techniques to Assess Intestinal Barrier Function In Vivo

### 6.1. Orally Ingested Probes Assessed in Urine

The current in vivo methods for permeability studies of the human intestinal mucosa cannot fully elucidate the passage routes. Hence, more detailed mechanistical studies of the intestinal barrier using the inhibitors/stimulators described above are not possible.

The first techniques used to investigate the integrity of the intestinal barrier function were in vivo permeability assays using orally ingested solutes excreted in the urine [96]. These early observations were followed by studies using inert probes of different sizes that are absorbed in various parts along the GI tract and excreted in the urine. By analyzing urine concentrations of the probes, the method provides an overview of the intestinal permeability and can be used both for assessment of small and large bowel permeability. This methodology was particularly used in early studies of small intestinal permeability in patients with IBD. The methodology involves the simultaneous use of small pore sized markers (5–8 Å) and large pore size markers (9.5–11 Å). The intestinal permeability is calculated as the ratio between the passage of the large pore and small pore marker, were the small pore marker represents the consistent flux across the mucosa [97]. By using the ratio intra-individual confounding factors are adjusted for [98].

The most common markers that have been previously used are the small pore markers; polyethylene glycols (PEG) 400 Da, the monosaccharides (mannitol and rhamnose) and the large pore markers; ^51^Chromium-ethylenediaminetetraacetic acid (^51^Cr-EDTA) as well as the disaccharides (lactulose, cellbiose) and PEG with a molecular weight of approximately 1000 Da [97]. Even though the in vivo permeability test does not distinguish between paracellular and transcellular permeability, the majority of large pore markers pass the mucosa via the paracellular route [99]. The in vivo permeability test using the lactulose/mannitol or lactulose/l-rhamnose ratio is frequently used to assess permeability of the small bowel [1,100]. An elevated flux of lactulose will generate an increased ratio between lactulose/mannitol or l-rhamnose and is a sign of loss of intestinal barrier integrity. Over the years the technique has become more sophisticated and studies are now performed using a multi-sugar test involving five different sugar probes; sucrose, lactulose, l-rhamnose, erythritol and sucralose [97].

The disaccharide sucrose is used as a marker of gastroduodenal permeability, while the ratio between erythritol and sucralose are used as assessment of colonic permeability.

Briefly, the in vivo permeability test is performed in fasted individuals that ingest a water mixture of the five sugars according to van Wijck et al. [97]. Urine is collected during the first 5 h in a continued fasted condition for assessment of small bowel permeability. During the following 5–24 h, urine is collected for assessment of colonic permeability under which participants are asked to avoid food and drinks containing similar sugars as in the multi-sugar mix. Collected urine samples are then analyzed for detection of the sugar probes using high-performance liquid chromatography (HPLC). Hence, the multi-sugar test gives an overview of the intestinal permeability but cannot be used to investigate the mechanistical pathways affected. Even though the test is non-invasive it can be perceived as complicated for certain individuals, and particularly the overnight fast and the collection of urine is often viewed as obstacles. Hence, good communication and support during the study is essential in order to secure compliance to the study protocol.

### 6.2. Biomarkers for Assessment of Intestinal Permeability

#### 6.2.1. Zonulin

During the last decade, a particular effort has been put on identifying reliable biomarkers able to assess intestinal permeability in blood. One of the first proteins identified with promising results was zonulin (47 kDa), an endogenous human analogue of the bacterial enterotoxin zonula occludens toxin. Zonulin has been proposed to modulate intestinal permeability by disassembling the tight junctional protein complexes in the intestinal epithelium [20]. Zonulin is a precursor to haptoglobin-2 and belong to the haptoglobin family of acute-phase reaction proteins [101]. Serum or plasma levels of zonulin has been suggested to mirror intestinal permeability and several conditions have been associated with increased zonulin levels [20,102,103]. Even though zonulin has emerged as a popular serological marker of intestinal barrier function caution should be taken when using commercially available assays. Recent studies further demonstrate that the assays currently available on the market do not detect zonulin (prehaptoglobin-2), but instead quantifies the levels of haptoglobin and complement factor C3 [104,105]. Hence, until the methodology has developed further, our recommendation is that one should interpret the levels of zonulin as a marker of barrier integrity with caution.

#### 6.2.2. Fatty Acid Binding Proteins (FABP)

FABP are approximately 15 kDa cytosolic proteins that bind and transport fatty acids. Several distinct types of FABP exists with different immunological functions depending on which tissue they are located in. Apart from the intestine, FABP are also found in heart, liver, muscle and adipocyte tissue [106]. In the intestinal enterocytes, largely in the absorptive part of the intestinal villus epithelium, both liver-type (L-FABP; FABP1) and intestinal-fatty acid binding proteins (I-FABP; FABP2) are expressed [107]. Intestinal FABP is only expressed in the intestine while LFABP can be found in liver and kidney [108]. In addition, ileal lipid or bile acid binding protein (ILBP or BABP; FABP6) is present in distal ileum where it has a high affinity for binding bile acids contrary to the other FABPs present in the intestine [109].

Given intestinal FABPs (L-FABP, I-FABP) high affinity for binding long chain fatty acids it has been implicated that they have a role in the intestinal absorption of lipids, as reviewed by Gajda AM et al. [107]. During intestinal ischemia and several diseases of the small intestine the intestinal epithelium is damaged and I-FABP is released to the blood stream and can be detected in plasma [110]. Plasma I-FABP has been found to correlate to mucosal injury in rats and pigs [106,110]. In humans I-FABP has been identified as a sensitive marker of intestinal ischemia [110,111]. Moreover, I-FABP levels were found to correlate with intestinal epithelial damage in a human ischemia-reperfusion model [112], and has also been identified as a diagnostic marker for complicated and uncomplicated necrotizing enterocolitis [113]. A recent study further identified that high serum levels of I-FABP in diarrhea predominant IBS patients correlate to an increased small intestinal permeability as assessed by the lactulose/mannitol ratio in urine [114]. Hence, I-FABP has emerged as a potential biomarker of intestinal barrier dysfunction [115].

#### 6.2.3. Citrulline

Citrulline is a non-protein amino acid produced mainly by the enterocytes of the small bowel with glutamine as a precursor [116,117]. Citrulline has been proposed to be a marker of reduced enterocyte mass [118]. A recent systematic review illustrates that citrulline correlates negatively with intestinal disease severity in enteropathies occurring in for example celiac disease and Crohn’s disease [119]. Hence, loss of small bowel epithelial mass is proposed to result in increased intestinal permeability. Circulating citrulline was found to decline in patients undergoing hemopoietic stem cell transplantation due to oral and GI mucositis, (leading to the loss of epithelial mass) as a result of intensive myeloablative therapy [118]. In an additional study the citrulline assay was further found to have higher specificity and sensitivity to small intestinal permeability than the in vivo multi sugar test in patients receiving myeloablative therapy [120]. However, as citrulline is a non-protein amino acid the level in plasma will be dependent on the absorption from food [121]. Citrulline is not commonly present in food except from watermelon (1 g citrulline/780 g), however, increased ingestion of watermelon during three weeks did not increase the plasma concentration of citrulline [122]. In autoimmune disorders such as rheumatoid arthritis, citrulline exists as citrullinated peptides through postranslationally modified arginine residues as reviewed by van Holers et al. [123]. Hence, caution should be taken when interpreting citrulline levels as a marker of intestinal permeability in autoimmune conditions [124].

#### 6.2.4. Glucagon-Like Peptide (GLP)-2

GLP-2 is a cleavage product of glucagon and a trophic factor specific to the bowel. It is secreted from the L-cells, an enteroendocrine cell, in the intestinal epithelium [125]. The main role of GLP-2 is proposed to be maintenance of growth and absorptive function of the intestinal villus epithelium as reviewed by Drucker and Yusta [126]. In mice, GLP-2 has shown to reduce paracellular transport of ions and small molecules and inhibit endocytic uptake of macromolecules [127]. Therefore, the reduction of GLP-2 might indicate a perturbed intestinal barrier function. Moreover, feeding obese mice with a prebiotic diet improved gut barrier function as well as reduced the levels of lipopolysaccharide (LPS) in plasma. These observations were associated with increased *bifidobacterium* and *lactobacillus* species in the gut microbiota and dependent on GLP-2 [128]. Hence, GLP-2 might be an important complement in intervention studies investigating the effect of pro- and prebiotic supplements on the intestinal barrier function. However, the food intake needs to be thoroughly monitored as well as the gut microbiota composition as the secretion of GLP-2 is stimulated by intake of common food components such as glucose, fatty acids and dietary fibers [129].

#### 6.2.5. LPS

Serum levels of the endotoxin LPS, present in the outer membranes of most gram-negative bacteria, have been implicated as a potential marker of an increased intestinal permeability, more specifically a marker of bacterial translocation [58]. Endotoxemia, i.e., the increased level of serum-LPS originating from the intestinal microbiota due to increased intestinal permeability has been linked to several diseases [130]. The hypothesis is that physiologic stressors such as dietary components, psychological distress or conditions causing a dysbiosis of the gut microbiota disrupt the intestinal barrier leading to increased permeability and subsequently an enhanced entry of bacteria and endotoxins into the systemic circulation that could contribute to systemic inflammation and further trigger numerous diseases [58]. The intestinal barrier has evolved to protect the body from harmless substances and direct interaction with the gut microbiota. Hence, toxicity only occurs when the barrier is broken and bacteria and bacterial endotoxins, such as LPS, reach the basal membrane of the enterocytes and underlying tissue. In most cases the immune system will handle the inflammation but in rare cases bacteria may reach the bloodstream and if they produce high amounts of LPS, septic shock may arise [131]. Endotoxins like LPS enter the cell mainly via lipid rafts and clathrin-dependent mechanisms and are then released from the cell through exocytosis at the apical cell membrane [132]. While exotoxins secreted by enteropathogenic bacteria have the ability to cross the intestinal mucosa the intact intestine of healthy individuals does not absorb most endotoxins produced by commensal bacteria [133]. The association of endotoxin levels to certain diseases, such as diabetes, has recently gained a large interest but is only based on measuring LPS in the blood or tissue of affected individuals. Moreover, small amounts of LPS (≤5 pg) have been detected in the blood stream of healthy individuals without causing any side effects [134]. A high fat diet have also been shown to temporarily increase LPS levels in blood in healthy individuals [135]. 

The methodologies used for LPS-detection in blood have been criticized for being imprecise and results have been contradicting [130]. LPS-levels vary considerable between methods and individuals and it is therefore difficult to interpret the significance of the detected LPS [136]. In addition, it is not evident if the LPS identified through the assays are bioactive and can cause a systemic inflammation and further contribute to the diseases they are associated with. Instead the heterogenous mixture of LPS might originate from bacterial cell walls or fragments bound to host proteins or associated with blood cells, as reviewed by Munford [130]. Hence, it is not possible to elucidate the relative abundance of LPS in blood and its stimulatory capacity of host cells in vivo. Moreover, LPS identified in blood may be derived from bacteria in other parts of the body, such as the oral cavity, or a local infection rather than the GI tract. Hence, LPS in plasma should be interpreted with caution as a measurement of a perturbed intestinal barrier and is preferably used in combination with other markers of intestinal permeability.

#### 6.2.6. LPS-Binding Protein (LBP)

Due to the difficulties in measuring and interpreting LPS, LBP has gained a lot of interest as a marker of the immune reaction towards LPS and hence as an indirect marker of endotoxemia [137]. LBP is an acute-phase protein produced by the hepatocytes that bind to bacterial LPS. The LPS-LBP complex further interacts with CD14, which promotes an inflammatory response cascade [138]. Levels of LBP in the systemic circulation have been associated with high-fat diets, obesity and IBD [137]. However, circulating LPB-levels vary during acute and chronic conditions and are affected by both diet and infection. Citronberg et al. showed that repeated measures of plasma-LBP are necessary to receive an acceptable reliability [137]. Although, LBP is more stable than LPS, increased levels of LPB only show that an immune response towards LPS has occurred in the blood. It will still not be possible to judge from where in the body the LPS originate.

#### 6.2.7. Fecal Markers of Intestinal Permeability and Markers of Intestinal Inflammation

One of the most abundant serine protease inhibitors in the circulation is alpha (α) −1-antitrypsin (AAT) [139]. It is primarily produced in the liver but is also secreted by diverse cell types as macrophages, enterocytes [140] and Paneth cells [141]. One of AATs main functions is to protect tissues from the proteolytic activity of immune cells, particularly neutrophils [139]. The AAT level is known to correlate to disease activity in Crohn’s disease and fecal AAT clearance is a marker of clinical disease severity in IBD [142]. In conditions with increased intestinal permeability due to disruption of the mucosal barrier AAT leaks from serum to the intestine. Due to its resistance to degradation by digestive enzymes in the gut, ATT has been used as a marker of intestinal permeability particularly in studies investigating permeability in environmental enteric dysfunction (EED) [143,144], liver disease [145] and lately in Parkinson disease [25]. Given the close relationship between intestinal inflammation and increased permeability markers of inflammation are often regarded as surrogate markers of intestinal permeability. Hence, AAT is often assessed together with fecal myeloperoxidase and calprotectin [143,144,146], as a measurement of neutrophil activity and subclinical intestinal inflammation [147]. Other markers of intestinal inflammation that could be assessed to complement the measurements of AAT and other in vivo permeability markers are fecal or serum lipocalin 2 (LCN2) [147,148,149,150] and serum amyloid A [151]. LCN2 is produced by intestinal epithelial cells, among other cell types [152], and is elevated in response to pro-inflammatory stimuli, like cytokines or Toll-like receptor activation [153]. The serum level of LCN2, in complex with metalloprotease 9, has been shown to correlate to disease activity in IBD [149,150] and is proposed as a surrogate marker of mucosal healing. Similarly, the acute phase protein amyloid A has been investigated in IBD [151]. Serum amyloid A is an acute phase protein produced in high levels in response to pro-inflammatory cytokines [154]. The increased levels were recently shown to correlate to the lack of mucosal healing in IBD patients [151] and have since then been considered a surrogate marker of mucosal inflammation. Although the close connection between inflammation and intestinal permeability, particularly in IBD, markers assessing primarily intestinal inflammation lie outside the scoop of this review. For further information on this topic the authors refer to [147] and [155].

In addition to the potential biomarkers mentioned above other markers have been investigated and suggested to have the potential to be used as serological biomarkers of intestinal barrier function. For example, the enzyme diamine oxidase which activity correlates inversely to intestinal permeability of the small intestine [156] has been proposed as a marker. Even though no biomarker so far is specific enough to determine a dysfunctional barrier and/or increased intestinal permeability on its own, serological biomarkers offer an important additional complement to other methodologies such as the in vivo multisugar test.

### 6.3. Assessment of Intestinal Permeability In Vitro

In contrast to the in vivo methodologies in vitro techniques offer possibilities to study mechanical processes of the intestinal barrier and individual cells in humans. Hence, more advanced studies using the previously described inhibitors/stimulators can be performed in these systems [157]. A majority of the basic knowledge of GI physiology has been achieved through in vitro techniques, and various methods for in vitro studies of intestinal mucosa have been developed.

#### 6.3.1. Caco-2 Cell Line

One of the most common cell lines used when studying intestinal barrier function are Caco-2 cells, due to the transport properties expressed [158]. The parental cell line has its origin in the colon and was developed from a human colon adenocarcinoma. Although being of colonic origin, the cells spontaneously differentiate into a polarized monolayer expressing several morphological and functional characteristics of the enterocyte. Hence, the cell line adapts common features of the small intestine [159]. This is characterized by the cylindrical polarized morphology of the cells with microvilli on the apical side and the formation of tight junctions between cells and the small intestinal ability to synthesize hydrolases, such as sucrose-isomaltase [159]. The exact feature of the Caco-2 cell line is dependent on the period of time the cells are cultured for, where a longer time generates more small bowel like features [160]. In addition, an increased number of cell passages lead to the selection of faster growing cells, which results in an augmented TER as well as an altered expression of several transporters important for intestinal barrier function [161]. Thus, it is essential to standardize growth condition, including cell density to obtain reproducible experimental models and comparable results. Certain clones have been isolated and characterized, in regard to brush boarder structure and transport activities, from the parental Caco-2 cell line to obtain a more homogenous population, as described by Sambuy et al. [161]. Once differentiated and grown to a confluent monolayer on transwell filter supports, as assessed by monitoring TER, the Caco-2 cell line offers a model of the small intestinal epithelium with the possibility to study epithelial-particle/bacteria/probe interactions and its effect on intestinal permeability [162,163].

#### 6.3.2. T84 Cell Line

Apart from Caco-2 cells, the T84 cell line is extensively used in research focusing on intestinal barrier function. The T84 cell line has, similarly to Caco-2, its origin in the colon and was derived from a lung metastasis of a colorectal adenocarcinoma. The cell line differentiates spontaneously into a monolayer and when confluent it displays structurally and functionally mature absorptive epithelial cells [164]. However, on the contrary to the Caco-2 cell line, T84 cells do not acquire small intestinal characteristics but retain much of their original colonic features throughout the differentiation process as described by Devriese et al. [164]. Thus, the T84 cell line is a better model for studies of the colonic barrier function compared to Caco-2.

#### 6.3.3. SK-CO15 Cell Line

The SK-CO15 cell line is derived from a human colon adenocarcinoma and forms a tight polarized epithelium with apical junctional complexes and domes when cultured on impermeable cell culture supports [165,166]. The SK-CO15 cell line resembles the colonic epithelium and lacks the common small intestinal differentiation markers, such as sucrose-isomaltase [166]. The SK-CO15 cells have particularly been described as a model system for studies investigating the Na^+^ absorption across the intestinal epithelium as the cells abundantly express the primary brush-border Na^+^/H^+^ exchanger type 3 [165]. In addition, the SK-CO15 cells have been used in studies investigating ethanol-induced barrier disruption [167] as well as when investigating the regulation of the intestinal barrier function via adherence and tight junctions [168,169].

#### 6.3.4. HT29 Cell Line

The heterogenous adenocarcinoma cell line HT29 undertakes a small bowel structure when differentiated, as described by Zweibaum et al. [170]. However, the HT29 cells cannot be entirely compared with absorptive enterocytes of the small intestine as the cells do not express all hydrolases and the ion transport properties are different compared to enterocytes present in the small intestine [171]. Initially, the HT29 cell line was used to study different aspects of human cancers but have lately attracted attention due to the fact that they are able to express different characteristics of intestinal epithelial cells. When grown under standard conditions the HT29 cells display an undifferentiated phenotype and do not express any typical characteristic of intestinal epithelial cells, as reviewed by Martínez-Maqueda et al. [171]. However, when these cells are grown in media where galactose replaces glucose, the cells can be modulated to produce mucin and the HT29-C18N2 clone has therefore been used as a model system for goblet cell differentiation [172]. Other mucin-producing clones have emerged from the parental cell line HT29 after subculture with sodium butyrate or 5-fluorouracil [173,174]. In contrast to other HT29 clones, the HT29-MTX cell line are induced to produce relatively high levels of mucin by stepwise adaptation to increasing concentrations of methotrexate (MTX) [173,175]. When grown on transwell filter supports, the HT29-MTX clones form a polarized monolayer where the majority of the cells are comprised of mature goblet cells secreting an adherent mucus layer [176]. Due to the ability to produce mucus, these HT29 clones are widely used as models to investigate the adherence of commensal and pathogenic bacteria to host cells [171,177]. Moreover, these cell-lines could be an asset in permeability studies as the mucus layer is an essential part of the intestinal barrier.

It is important to note that exchanging the media to galactose generates a heterogenous population of HT29 cells with both secretory and absorptive cell types [172]. This may, depending on the type of experiment being performed, challenge permeability studies. The HT29 clone, HT29cl.f8, is derived from a single cell of HT29 and spontaneously polarizes under standard conditions in glucose and display important characteristics for permeability studies such as microvilli, tight junctions and a high TER [178]. This particular clone, can therefore be an alternative and have been used in several studies to model the intestinal barrier [179,180].

#### 6.3.5. Co-Culture of Cell Lines

Even though separate cell lines can be easily established it is important to view them as a simple model of the intestinal epithelium as they lack the contact with immune cells and signals from nerves and luminal stimuli which is the normal condition in the intestine and proven lately to have a great impact on intestinal barrier function [42]. To establish more realistic in vitro models to mimic the environment in the intestine several co-culture models and even triple-cultures have been established, various co-culture models, representative of the small intestine, have been developed over the years [181]. A mucus-producing model of the intestinal epithelium is often established by co-culturing Caco2-cells with HT29-MTX clones [182,183]. Different ratios of the two cell lines in the co-culture have been previously explored to create a condition representing the physiology most relevant to the in vivo environment of the small intestine mimicking the different parts of the intestine. The co-culture grows into a monolayer with a continuous mucus layer and has been used in intestinal permeability studies [184,185]. One limitation of these models is that they do not develop crypts and villus structures observed in the small intestine as they are grown in monolayers. Recently, Chen et al. [186], developed a triple culture model where a three-dimensional (3D) porous silk scaffolding tube was engineered and coated with a culture of Caco-2 and HT29-MTX epithelium. Primary human intestinal myofibroblasts were grown in the tube scaffold space underneath to stimulate epithelial growth and differentiation [186]. In this model a crypt like structure, as observed in the small bowel, was formed. The 3D model further showed an increased production of MUC2 compared to transwell co-cultures. Even though cell function was observed to decline after a few weeks of culture, these systems offer a model with many physiological similarities to the small intestine particularly useful for short-term studies regarding bacterial-epithelial interaction [186] and potentially intestinal permeability studies. Interestingly, a long-term 3D model of the small intestinal epithelium was recently established by Dosh et al., using Caco-2 and HT29-MTX cells co-cultured on a hydrogel (L-pNIPAM) scaffold [187]. This model was particularly developed to facilitate long-term mechanistic studies in an inflammatory environment, similar to that in IBD [188]. Future research will have to elucidate how bacterial translocation experiments can be performed using models grown on different scaffolds.

In addition, a co-culture model was recently established using Caco-2 cells and differentiated THP-1 monocygotic cells to facilitate studies of the intestinal barrier in a healthy and inflamed state [189]. A stable co-culture model resembling the healthy state in the intestine was established by co-culturing Caco-2 cells and THP-1 cells for 48 h. An inflamed short-term condition was induced by priming the Caco-2 cells with IFN-γ, while the THP−1 cells were pre-stimulated with LPS and IFN-γ. This resulted in a temporary reduction in barrier integrity through the measurement of TER and release of elevated levels of pro-inflammatory cytokines. As the model is comprised of epithelial cells and macrophages it offers a possibility to perform experiments investigating the effect of toxicants, bacteria and other substances on the intestinal barrier [189].

An important feature of the small bowel is the follicle-associated epithelium where the M cells are located which are specialized in the sampling of antigens and foreign substances in order to establish immune tolerance but also to launch an appropriate immune response towards pathogenic bacteria [190]. Kernéis et al. [191] showed that co-culture of Peyer’s patch B-cells and intestinal epithelial cells provoke the development of an M cell-like phenotype and since then, modifications of the model have been established [192,193]. As a continuation, a triple co-culture model including co-culture of Caco-2 and HT29 cells followed by the addition of Raji B-cells, was developed [194,195]. This model was particularly developed for advanced studies of drug absorption and showed an increased permeability of insulin in the triple co-culture models compared to the Caco-2/Raji B cell model, indicating, that the presence of goblet cells in the triple culture may influence drug transport across the intestinal barrier. Future studies need to elucidate how this model could be used when investigating bacterial translocation.

### 6.4. Intestinal Organoids as a Model to Assess Barrier Function

In the last decade there has been a particular focus on the development of techniques making it possible to maintain intestinal epithelial cells (IEC) in vitro as intestinal organoids. When cultured in novel systems the IECs recapitulate the physiology of the 3D structure and genetic signature of the original intestinal epithelium and forms intestinal organoids [196], where all differentiated cells (i.e., absorptive enterocytes, goblet cells, enteroendocrine cells, Paneth cells, tuft cells and M cells) comprising the intestinal epithelium are present. This is a clear advantage compared to intestinal epithelial cell lines and makes intestinal organoids a very interesting and useful model for studies regarding intestinal barrier function, as reviewed by Nakamura [197]. The differentiated cells of the intestinal epithelium are derived from multiple lineages that originate from intestinal stem cells (ISC) [198]. The ISC reside at the bottom of the crypts of the intestinal epithelium [199,200,201] and are capable of self-renewal and multi-lineage differentiation.

Sato et al. [202] isolated crypt cells from the mouse small intestinal epithelium, embedded them in matrigel and let the cells grow for an extended period and observed that the cells self-organize into 3D structures, organoids, of the intestinal epithelium in the presence of growth factors. Currently several different methodologies to culture organoids from other intestinal cells have developed and it is now possible to generate organoids from colonic cells, fetal intestinal progenitor cells of both rodents and humans [203,204,205,206,207,208]. The methodology is further used to establish organoids from patients suffering from different diseases, such as celiac disease [209] and IBD [210]. Intestinal organoids grown in a 3D structure offers an ideal model to study the different functions of specialized cells in the intestinal epithelium. For example, the function of goblet cells in cystic fibrosis [211], the control of the degranulation by Paneth cells [212] as well as the function of the more recently discovered tuft cells [213,214,215] are all examples of studies performed using intestinal organoid systems. Moreover, intestinal organoids offer an exciting model to study intestinal barrier function and host-microbe interactions [197]. The model have for example been used to investigate the effect of *Salmonella* infection on IECs [216] as well as the effect of *Clostridium difficile* on epithelial barrier function [217]. Nevertheless, it is difficult to perform large experiments with simultaneous stimulations of 3D intestinal organoids as it requires microinjection of the substance or bacteria of interest to reach the inner surface of the intestinal organoid. Two-dimensional systems of intestinal organoids grown on transwell inserts [218,219] have therefore been developed to facilitate studies of host-microbe interactions, functional studies of the intestinal barrier as well as drug discovery [197,218,220]. In addition, studies have been performed where intestinal organoids are used for thorough studies of the intestinal barrier using the ex vivo Ussing chamber [221,222] as described in detail below. Although intestinal organoids are an exciting model to use for studies of the intestine and intestinal barrier function it was recently demonstrated that organoids established from inflamed tissue lost parts of their inflammatory characteristics after 1 week of culturing and after 4 weeks they could be distinguished from organoids established from non-inflamed tissue [223]. However, recent findings indicate that the inflammatory state can be re-induced with different inflammatory mixtures independent of the state of the tissue origin [224]. Hence, depending on length and aim of the experiment it might be important to consider this when setting up organoids as a patient-specific intestinal model.

### 6.5. Assessment of Intestinal Barrier Function Ex Vivo—The Ussing Chamber Technique

The Ussing chamber technique, first described in 1951 by the Danish physiologists Ussing and Zerhan [225], has many applications, there among studies on ion transport, drug absorption, protein absorption, and several pathophysiological processes in both animals and humans [225,226,227]. Therefore, offering an advanced system allowing thorough mechanistic studies of the intestinal barrier through the use of inhibitors and stimulators. This methodology is today the most advanced one that thoroughly assesses intestinal barrier function; however, it is important to acknowledge that the tissue is removed from its natural context and therefore lacks the contact to nerves and luminal content. The initial methodology of the Ussing chambers was rather complicated, and the methodology was later modified and simplified by Grass and Sweetana [228]. The general principle is that a flat sheet of mucosa is mounted between two half-chambers filled with continuously oxygenated buffer. The permeability probes, described later, are added to the buffer of the mucosal chamber and after defined time intervals, samples are collected from the serosal chamber. One pair of electrodes gives current to the system while one pair of reference electrodes with agar salt bridges enables the monitoring of electrophysiological parameters. The permeability markers/probes are added to the buffer of the half-chamber of where the mucosal side of the tissue is facing, the so-called mucosal chamber. After defined time intervals, samples are collected from the chamber where the serosal side of the tissue is facing, the so-called serosal chamber, as a measurement of passage. The good viability-supporting possibilities with oxygenation and effective circulation of the fluid on both sides of the tissue, combined with the possibility to monitor membrane electrophysiological parameters, provide the Ussing chamber technique with important advantages compared to other in vitro techniques for intestinal tissue experiments [226]. During the experiment, chambers are kept at 37 °C and two pairs of electrodes enable the monitoring of electrophysiological parameters, i.e., the potential difference (PD), short circuit current (Isc) and TER, which verifies tissue viability throughout the experiments.

The characteristic for all epithelia is the ability to maintain a PD, and the ability depends on the electrogenic ion pumps activity in the epithelial cell membrane. The current needed to nullify the PD is defined as Isc, which is a function of the ion pumps activity. The TER reflects the electrical resistance of the paracellular routes, mainly via the tight junctions. For more details on the Ussing chamber technique and how it can be set up, authors refer to [229,230].

## 7. Permeability Markers

Tight junction alterations are readily indicated by the changes of TER [231], however, in order to study intestinal permeability and integrity of tissues or cell culture models, measurements of permeation markers of different sizes need to be applied. Depending on size, the markers are used as paracellular or transcellular probes in in vitro and ex vivo studies. Bacteria can be used not only as permeability markers, but also to study interactions with the epithelium.

### 7.1. Paracellular Probes

#### 7.1.1. ^51^Cr-EDTA

The EDTA molecule is known to pass between the cells via the paracellular route. The binding of EDTA to the radioactivity labelled Cr is extremely strong, which assures that the passage of Cr is equal to the passage of EDTA, and no Ca^2+^ can bind to EDTA to give detergent effects. The inert probe ^51^Cr-EDTA with a molecular weight of 384 Da is often utilized in vivo since it is stable in the colonic luminal environment allowing assessment of colonic permeability and it is easily detected in the urine by gamma-counting. Numerous studies have evaluated intestinal paracellular permeability using ^51^Cr-EDTA, in both an in vivo [232,233] and ex vivo setting [11,229].

#### 7.1.2. Fluorescein Isothiocyanate (FITC)-Dextran

An alternative to radiolabeled probes is markers conjugated to fluorochromes. FITC-dextran 4000 is a fluorescently labelled sugar molecule of 4 kDa. FITC-dextran 4000 is a well-documented paracellular probe and is widely used to study intestinal permeability in in vitro [145,176]. The advantage of using FITC-dextran 4000 compared to using for example ^51^Cr-EDTA is obviously that it is not radiolabeled which makes it easier to the use. Passage experiments of biopsies mounted in Ussing chambers have confirmed that FITC-dextran 4000 and ^51^Cr-EDTA provide equal results of paracellular permeability [234].

#### 7.1.3. PEG

Polyethylene glycols are polymers that can be found in different sizes (for example 400, 600, 900, 1000, 3000, 4000 Da) and are analyzed by mass spectrometry. In contrast to other paracellular probes, that provide limited information on size-dependent changes in permeability, the method including PEG of different sizes can also measure the size-dependence of apparent permeability. Experiments using PEG of various molecular weights thereby provides a probing of the functional regulation of the paracellular route and are an important tool to determine both the pore and leak paracellular pathway [235,236]. As mentioned above, PEG of 400 and 1000 Da are common probes in in vivo experiments.

#### 7.1.4. ^14^Carbon (C)-Mannitol and ^14^C-Inulin

Mannitol is a hydrophilic 182 Da sugar molecule that permeates across epithelial barriers through the aqueous pores in the tight junction complexes and is therefore used as a paracellular permeability marker [237,238]. Inulin is another sugar molecule which crosses through the barrier comparable to mannitol [239]. Mannitol or inulin labelled to ^14^C can easily be detected in a gamma-counter, similar to ^51^Cr-EDTA.

#### 7.1.5. Lucifer Yellow

Lucifer yellow is a fluorescent molecule with a size of 444 Da that is known to pass between the cells via passive diffusion. It can be detected in fluorescence microscope for uptake studies, and by fluorimetry for passage studies (excitation wavelength 424 nm/emission wavelength 525 nm). Lucifer yellow has been used for studies of paracellular permeability in both in vitro [216,240] and in vivo [230,241] settings, and especially in studies of drug absorption.

#### 7.1.6. Biotin-Labelled Probes

One way to visualize paracellular passage of macromolecules is by using biotin-labelled probes. In 2016, Richter et al. developed a new method for imaging of paracellular passage sites [242] and this method was recently modified by Krug et al. [243]. This sandwich assay relies on the avidin–biotin system and includes biotinylated and fluorescent-conjugated dextrans that bind to the basolateral membranes of the epithelial cells that are pre-labelled with cell-adherent avidin. The probe solution can be either one single biotinylated dextran or a sequence of biotinylated dextrans of various sizes and fluorescent labels.

### 7.2. Transcellular Probes

#### 7.2.1. Horseradish Peroxidase (HRP)

HRP is a 45 kDa protein antigen used as a marker of protein uptake with the antigenic potential to initiate immune responses in humans. Under normal circumstances, HRP is known to pass through the cells via macropinocytosis [244,245]. HRP is easy to detect by ELISA and has been frequently used for permeability studies in Ussing chambers [42,229,246]. One advantage of HRP is its possibility to be detected by electron microscopy [229,247].

#### 7.2.2. Fluorescent Labelled Particles and Bacteria

The uptake and passage of particles have been studied in vitro in various cell models, but also in Ussing chambers. In most studies, fluorescent latex (polystyrene) or polymeric poly-lactid-co-glycolidic acid particles have been used [248,249]. The fluorophore can either be attached to the particle surface or incorporated into the particle. Latex particles can be found in many dyes and defined sizes, depending on the purpose. For example, sizes of 0.5–1 μm might be optimal for transcellular in vitro studies of cells cultured on transwell filters, while sizes of 2–5 μm might be optimal for ex vivo Ussing chamber experiments. It is therefore of importance to carefully consider the optimal particle size before implementing the barrier function experiment.

Bacteria can be added as a marker of transcellular bacterial uptake in a host or in vitro setting. Experiments can be performed using live or dead bacteria. A large number of fluorescently labeled dead (heat- or chemically killed) bacteria exist in a wide variety of sizes, shapes, and natural antigenicity. Killed bacteria have generally been used for studies of phagocytosis in the intestinal epithelium [250]. These fluorescent BioParticles^®^ (Molecular Probes, Leiden, The Netherlands) are for example available as strain *E. coli* K-12 which is found in many different wavelengths. Chemically-killed *E. coli* K-12 are killed with paraformaldehyde in such way that it stops their reproduction but retains their antigenicity, and has previously been used in studies of intestinal barrier function [229,251,252]. For transcellular studies in vitro and in Ussing chambers, a size of 0.8 × 0.2 μm is preferable. Passage of fluorescence-conjugated *E. coli* can easily be detected by fluorimetry or flow cytometry. An advantage of the chemically killed bacteria is that they are not affected by the oxygenated environment in the Ussing chamber/in vitro model, however, for thorough assessment of host-microbe interactions live bacteria are preferable.

When using live bacteria, it is important to consider the growth conditions of the bacteria that will be investigated. As both in vitro and ex vivo methodologies involve oxygenation in order to ensure the survival of cells/tissues it is important to carefully monitor how anaerobic bacteria survive during these conditions and also take the time factor into consideration. To be able to visualize uptake of live bacteria into the intestinal epithelium, bacteria are usually incorporated with green fluorescent protein (GFP). This methodology can be used both to study commensal bacteria, such as *E. coli* K12, *E. coli* HS and *E. coli* HB10 [6,229,251] but also pathogenic bacteria such as *Salmonella typhimurium* and *Yersinia pseudotuberculosis* [11,162]. Before usage, live bacteria have to be pre-cultured and diluted to a fixed CFU/mL, for example when using them for studies of intestinal tissues the concentration should be 1.0 × 10^8^ CFU/mL, to mirror the concentration of bacteria in the lumen. Passage of live GFP-incorporated bacteria can easily be detected by fluorimetry or flow cytometry. For investigation of host-microbe interactions, live bacteria can be added to the Ussing chamber for thorough assessment of how a specific bacterium or a group of bacteria affects the intestinal barrier and track the pathways and how the bacteria interacts with underlying mucosal cells.

## 8. Concluding Remarks

**Figure 2:**
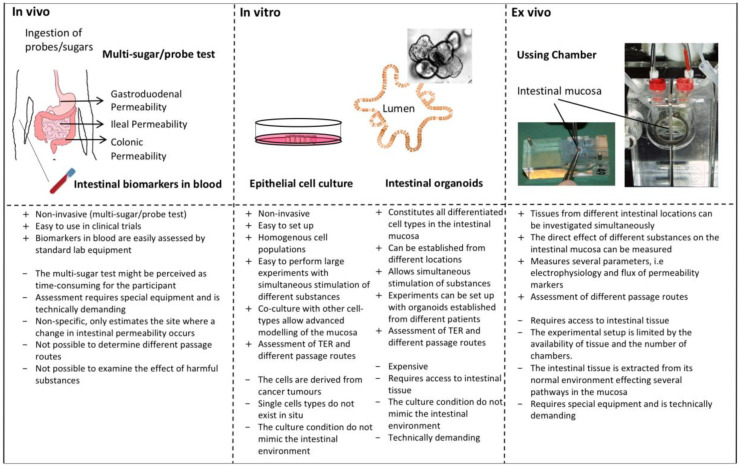
Overview of the different techniques used to measure intestinal barrier function. It is important to consider the aim of the study and the resources in the laboratory prior choosing the methodology. For a thorough assessment of intestinal barrier function the techniques are preferably combined, for example in vivo and ex vivo techniques can be combined with in vitro studies for a more mechanistic approach. TER: transepithelial resistance.

The interest for functional studies of the intestinal barrier has increased over the last years and new research continues to emphasize the central role of a leaky gut. Hence, the interest to perform these types of studies will most likely increase. As outlined in this review there is no gold standard to measure intestinal barrier function. Therefore, it is important to combine different techniques in order to give an accurate picture of the intestinal barrier as possible. Depending on how central the effects on the intestinal barrier is to the study aim, different methodologies can be used and combined as outlined in Figure 2.

This overview of current techniques available might make it easier for those new to the field to choose and combine methodologies to answer research questions regarding the intestinal barrier. It is however important to note that new approaches are constantly developing in this expanding field.

## Figures and Tables

**Table 1:** Major diseases and conditions associated with an increased intestinal permeability.

Disease/Condition	Paracellular Permeability	Transcellular Permeability	Uncategorized Permeability Changes
Inflammatory bowel disease	in vivo; altered expression and distribution of tight junction proteins [5]; ex vivo, increased passage of paracellular probes [6,7]	ex vivo; augmented mucosal passage of bacteria and horseradish peroxidase (HRP) [6,7,8]	in vivo; increased urinary secretion of probes [9]
Irritable bowel syndrome	altered expression of tight junction proteins [10]; ex vivo; increased passage of paracellular probes [1,2].	ex vivo; increased transepithelial passage of bacteria and HRP [11]	in vivo; increased urinary secretion of probes [12]
Celiac disease	in vivo: altered structure of tight junction proteins [13,14] ex vivo; increased passage of paracellular probes [15]; alteration in electrophysiological parameters [16]	ex vivo; augmented internalization of bacteria [17]; increased transcellular uptake of gliadin [18]	in vivo; increased urinary secretion of probes [15,19], increased levels of zonulin in blood [20]
Obesity	in vivo; altered expression of tight junction proteins [3]	ex vivo; increased lipid-induced transcellular permeability [3]	in vivo; increased levels of zonulin and lipopolysaccharide (LPS) in blood [3]
Diabetes type 2	-	-	in vivo; increased urinary secretion of probes [4]; increased levels of LPS [21] and zonulin [22] in blood
Alzheimer’s disease	-	-	in vivo: increased LPS [23] and zonulin levels in blood [24]
Parkinson disease	-	ex vivo: augmented uptake of bacteria [2]	in vivo: increased urinary secretion of probes [2]; increased blood zonulin levels [25]
Major depression disorder	-	-	in vivo: increased permeability markers in blood, I-FABP and zonulin [26]
Autism spectrum disorders	in vivo: altered expression of tight junction proteins [27]	-	in vivo: increased levels of zonulin [28,29]
